# Stress-Induced Takotsubo Cardiomyopathy Identified by Unique Nuclear Perfusion Pattern

**DOI:** 10.7759/cureus.103364

**Published:** 2026-02-10

**Authors:** Marc T Zughaib, Brittni McClellan, Emily Anton, Mahmoud Assaad

**Affiliations:** 1 Cardiology, Henry Ford Providence Hospital, Southfield, USA; 2 Research, University of Michigan, Ann Arbor, USA; 3 Interventional Cardiology, Henry Ford Providence Hospital, Southfield, USA

**Keywords:** cardiac imaging, cardiology, multimodality cardiac imaging, nuclear cardiology, takotsubo

## Abstract

Takotsubo cardiomyopathy (TC) is a stress-induced, reversible left ventricular dysfunction syndrome. Historically diagnosed via echocardiography and coronary angiography, nuclear imaging techniques have added utility and enhanced diagnostic accuracy. We present a case of a 49-year-old female who presented with dyspnea and was diagnosed with TC with the aid of nuclear myocardial perfusion imaging (MPI), which was confirmed with invasive coronary angiography.

Upon arriving at the ED, the patient’s ECG demonstrated a normal sinus rhythm with nonspecific ST changes. An echocardiogram indicated a cardiomyopathy with an LVEF of 30-35%, hyperkinesis of the base, and hypokinesis of the apex. A nuclear stress test revealed a large, severe fixed defect in the circumferential apical territories, with the bases spared, raising suspicion for TC.

The patient underwent cardiac catheterization, which demonstrated non-obstructive coronary artery disease. LV gram confirmed hypercontractility of the basal segments with akinesis of the apex, consistent with TC. She was initiated on goal-directed medical therapy with full resolution of her wall motion abnormalities on her follow-up visit.

TC can mimic acute coronary syndrome (ACS), and coronary angiography is the gold standard for differentiating these entities. Nuclear imaging can aid clinicians between ACS and TC by demonstrating an apical defect that does not correlate with a coronary artery territory.

## Introduction

Takotsubo cardiomyopathy (TC), also known as stress-induced cardiomyopathy, is characterized by transient left ventricular systolic dysfunction with basal hypercontractility and apical hypokinesis. It predominantly affects postmenopausal women and is often triggered by acute emotional or physical stress, leading to a catecholamine surge hypothesized to cause myocardial stunning [1]. Clinically, TC mimics acute coronary syndrome (ACS) with symptoms such as chest pain, dyspnea, and ECG changes but occurs without obstructive coronary artery disease [1]. This overlap complicates timely diagnosis.

Echocardiography remains the first-line imaging modality due to its ability to reveal characteristic wall motion abnormalities [2]. However, it cannot reliably differentiate TC from ischemic cardiomyopathy. Coronary angiography is the gold standard to exclude obstructive disease, but it is invasive. Nuclear myocardial perfusion imaging (MPI) can be a valuable non-invasive modality to aid in the diagnosis. MPI can provide additional information by demonstrating an apical perfusion defect inconsistent with a single coronary artery territory [3-5]. MPI visualizes myocardial blood flow rather than just mechanical function, thus providing complementary diagnostic information [5]. Here we present a case of TC, who was unusually diagnosed with MPI and confirmed with coronary angiography. This case underscores the importance of nuclear imaging in suspected TC and advocates for its inclusion in diagnostic criteria.

This case was previously presented as a meeting abstract at the 2025 National ACC Scientific Meeting on March 30, 2025.

## Case presentation

A 49-year-old woman with a history of tobacco use (0.5 packs per day for 10 years) presented with progressive dyspnea over several days. She had never experienced symptoms like this previously, occurring with minimal exertion. She had recently had an argument with her spouse, which preceded her symptoms. She had no other relevant cardiovascular comorbidities. Upon presentation, her blood pressure was 121/86, heart rate was 106 beats per minute, and oxygen saturation of 94% on room air. There was mild thrombocytopenia with a platelet count of 86 K/mcL on the complete blood count, and high-sensitivity troponin was initially 26, followed by an undetectable level <6 ng/L (Table 1).

**Table 1:** Laboratory studies upon presentation to the emergency department. Complete blood count, basic metabolic profile, high sensitivity troponin, and nt-ProBNP listed above with reference values. Labs not shown were within normal limits.

Lab	Value	Reference Range
WBC (K/mcL)	10.37	4-11
Hemoglobin (gm/dL)	13	12-16
Platelets (K/mcL)	86	150-400
BUN (mg/dL)	19	6-20
Creatinine (mg/dL)	0.8	0.5-1.0
Sodium (mmol/L)	135	136-145
Potassium (mmol/L)	3.7	3.5-5.1
Chloride (mmol/L)	101	98-107
CO2 (mmol/L)	23	22-29
Troponin - hs (ng/L)	26	0-10
nt-Pro BNP (pg/mL)	51	50-192

**Figure 1:**
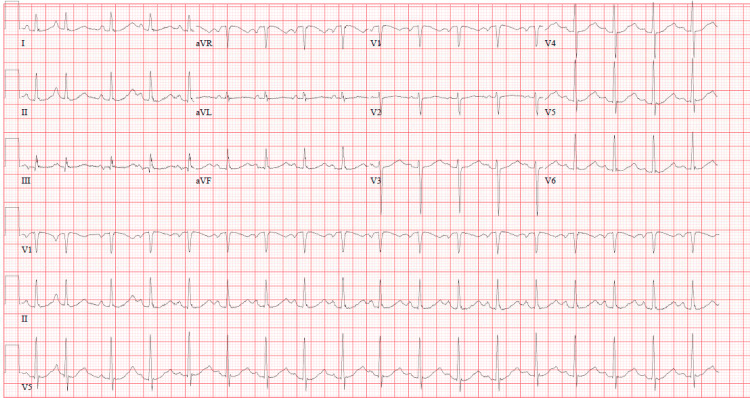
Presenting ECG demonstrating sinus tachycardia and an occasional premature atrial contraction.

Otherwise, laboratory evaluation (complete blood count, basic metabolic profile, and nt-ProBNP) were largely unremarkable.The thrombocytopenia was initially attributed to the patient's smoking history. The mild troponin elevation can be seen in cardiomyopathies. The troponin was then undetectable, making acute coronary syndrome less likely. ECG showed sinus tachycardia with a premature atrial contraction (Figure 1).

**Figure 2:**
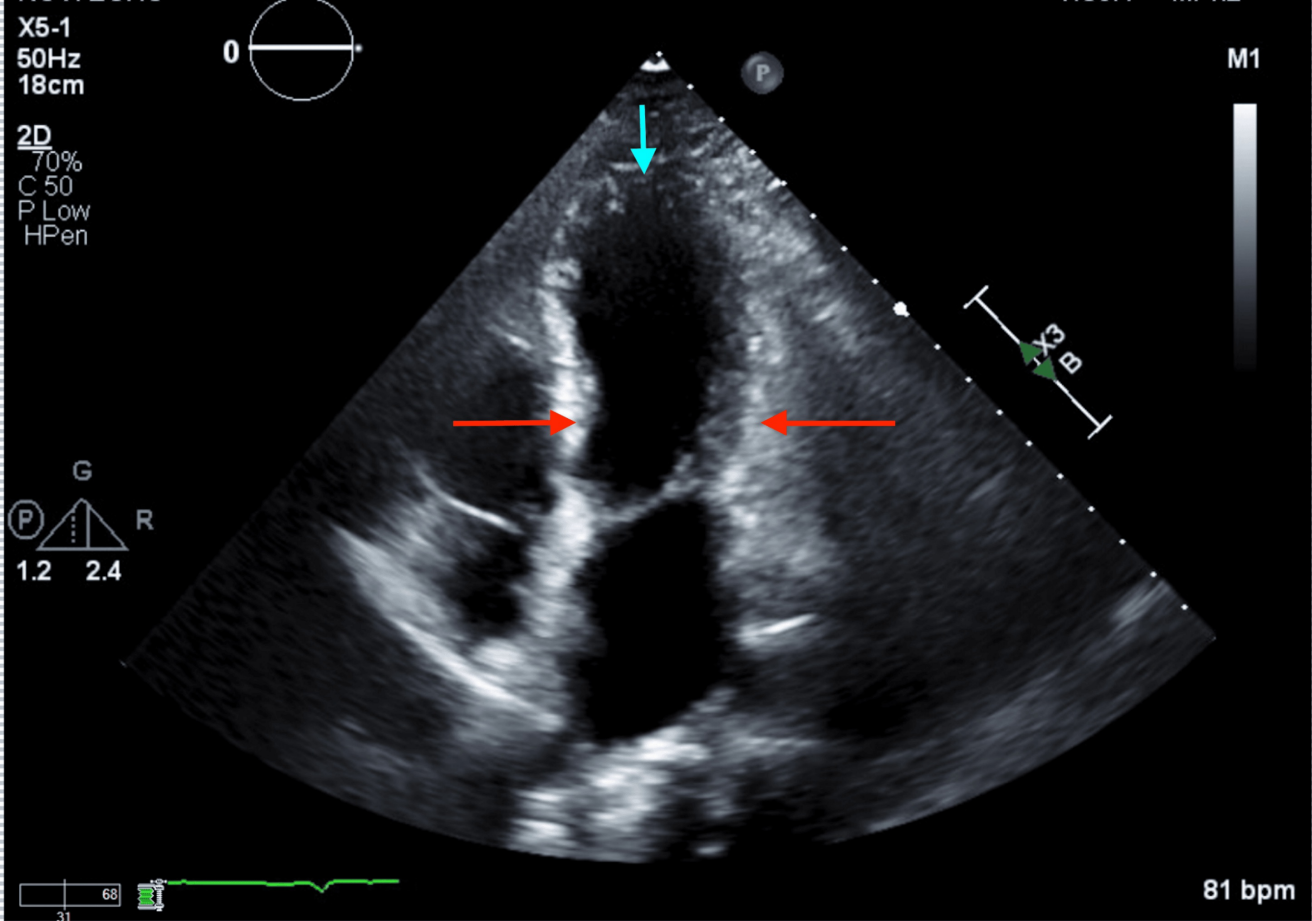
Apical four-chamber view on transthoracic echocardiography with hyperkinesis of the basal segments (red arrows) and apical ballooning (light blue arrow) concerning Takotsubo cardiomyopathy.

Physical examination revealed bilateral lung crackles and mild lower extremity edema. Transthoracic echocardiography demonstrated a cardiomyopathy with a left ventricular ejection fraction (LVEF) of 30-35%, hypokinesis of the mid to distal anterior wall, anterior septum, apex, and apical lateral wall, and hyperdynamic basal segments. Left ventricular size and wall thickness were normal (Figure 2).

**Figure 3:**
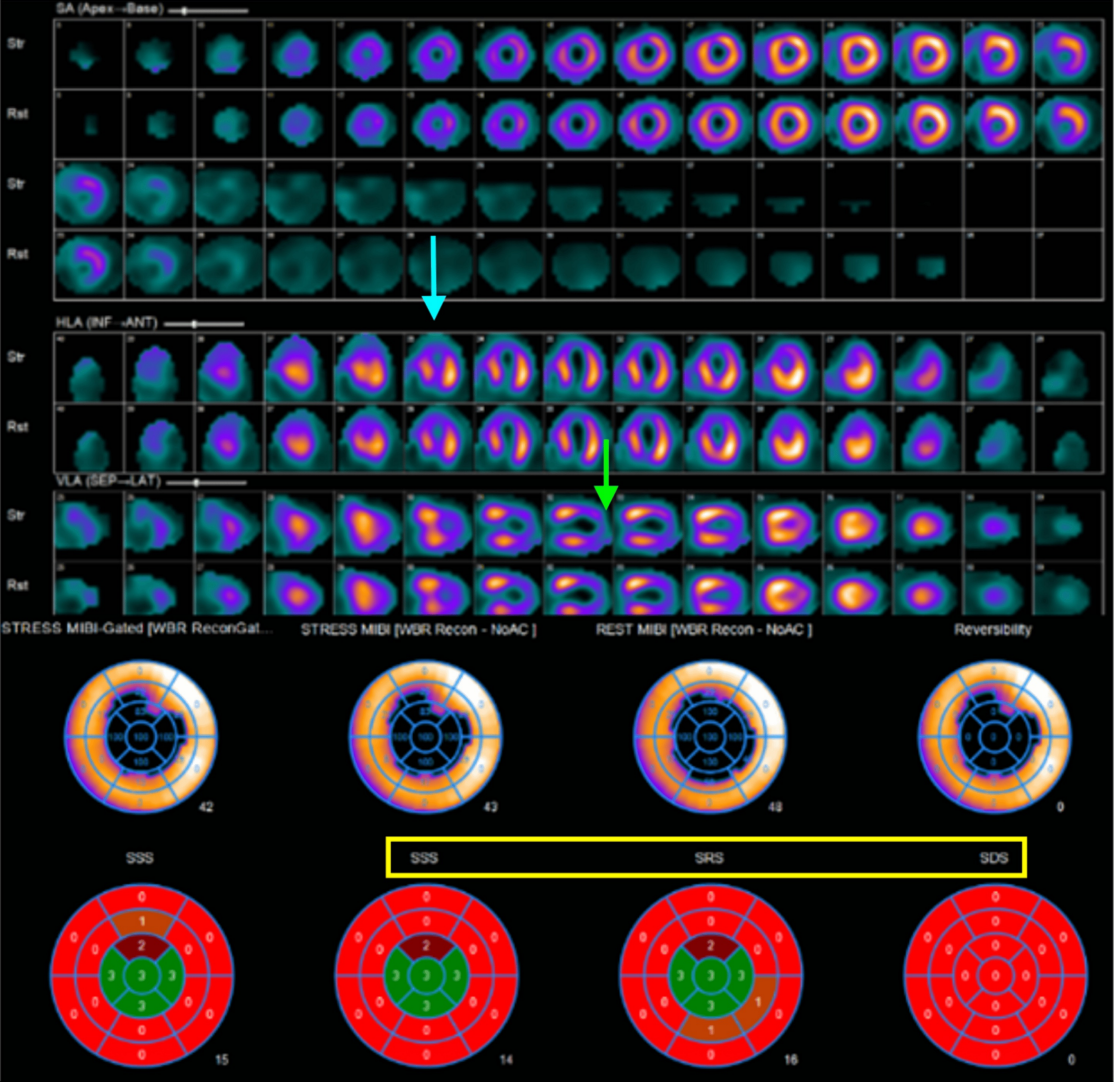
Myocardial perfusion imaging demonstrating large sized, severe intensity, fixed defect involving the apical segments. The horizontal long images (light blue arrow) and vertical long images (light green arrow) demonstrate the fixed defect at the apex. The stress (SSS) and rest (SRS) images at the bottom of the figure both show decreased perfusion at the apex (yellow boxes). On cine images (not pictured), there is hypercontractility of the basal segments with apical ballooning.

A pharmacological myocardial perfusion stress test with the vasodilator regadenoson was ordered. This decision to perform MPI rather than conventional coronary angiography was made as the suspicion of significant obstructive epicardial coronary artery was low due to the patient's relatively young age and lack of anginal symptoms. MPI provides a non-invasive modality without the potential risks of vascular or hemodynamic adverse events associated with conventional coronary angiography. The MPI was performed, and this study demonstrated a large-sized, moderate-to-severe intensity, fixed perfusion defect circumferentially involving the mid to distal apical territory, sparing basal segments (Figure 3).

Upon review of the functional images in the MPI, there was hypercontractility of the basal segments with hypokinesis/apical ballooning of the apex. At this time, the differential includes a high-grade stenosis in the left anterior descending artery that wraps around the apex, or TC. This perfusion pattern is highly suggestive of TC, as defects do not align with a single coronary artery territory [3,4]. 

**Figure 4:**
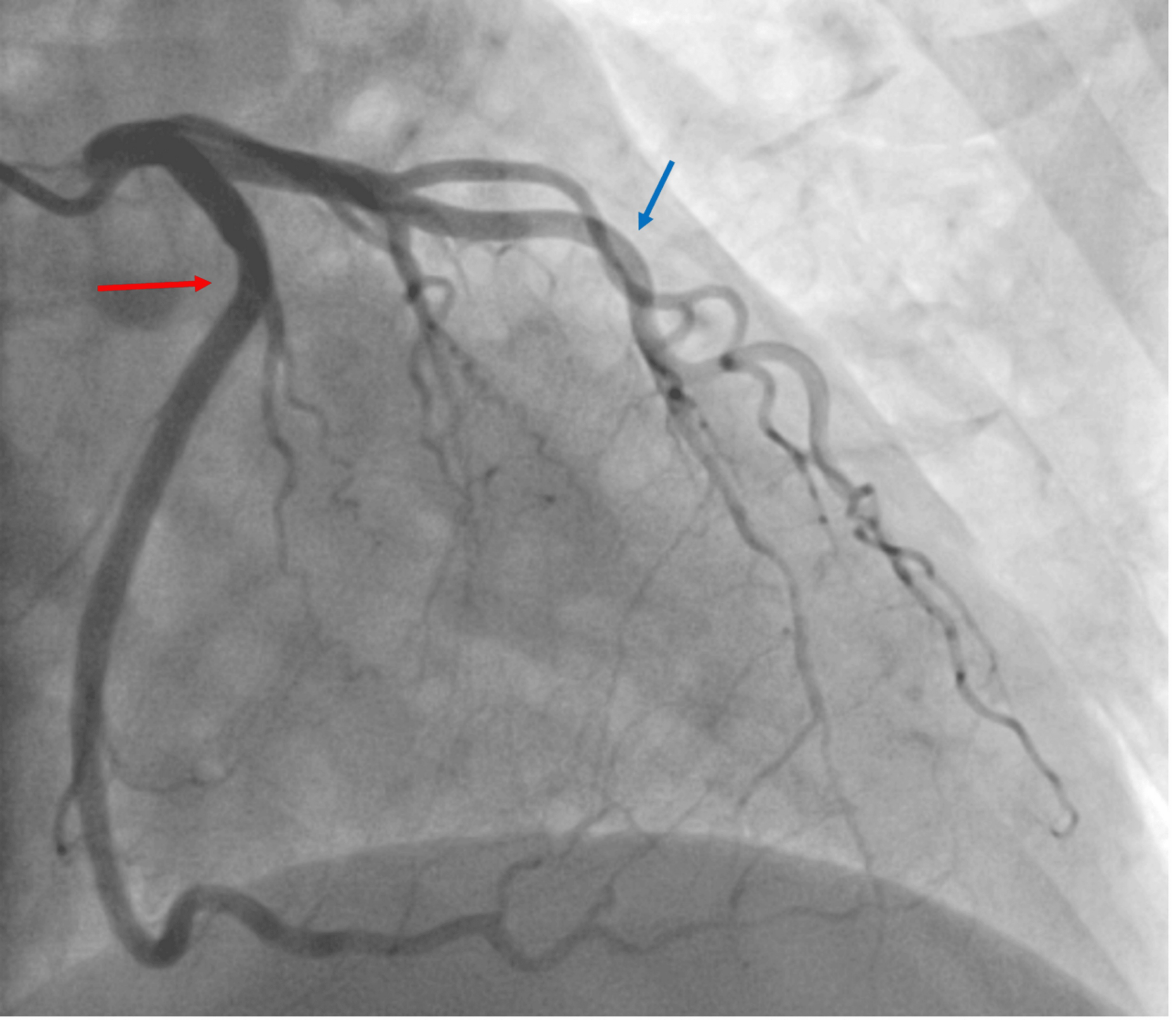
Right anterior oblique cranial view of the coronary arteries during left heart catheterization. Coronary angiography revealed non-obstructive coronary artery disease with minor luminal irregularities of the left anterior descending artery (blue arrow) and a large circumflex (red arrow) without coronary artery disease.

The next diagnostic step required invasive coronary angiography after the MPI demonstrated a large-sized perfusion defect. Coronary angiography revealed non-obstructive coronary artery disease with minor luminal irregularities of the left anterior descending artery. There was no epicardial coronary artery disease that would explain the wall motion abnormalities or perfusion defect. Left ventriculography was subsequently performed, which confirmed basal hypercontractility with apical akinesis, consistent with TC. Left ventricular end-diastolic pressure was mildly elevated (16 mmHg) (Figure 4). 

## Discussion

This case illustrates the diagnostic complexity of TC, which can mimic an acute coronary syndrome presentation, but requires distinct management from this entity. Our patient's emotional stress after an argument with her spouse was determined to be the inciting event for her presentation. She was able to recover her left ventricular function after tolerating medical therapy for 3 months. The use of non-invasive imaging aided in the diagnosis, which was ultimately confirmed with coronary angiography. 

Echocardiography is critical to the diagnosis, but cannot conclusively differentiate TC from ischemic cardiomyopathy [2]. The diagnosis of TC on nuclear myocardial perfusion imaging (MPI) is exceptionally rare, highlighting the unique nature of this case. According to the European Association of Cardiovascular Imaging and Japanese Society of Echocardiography joint consensus document, "the role of nuclear imaging in TTS has not yet been well established," underscoring that nuclear imaging is not routinely employed in the diagnostic workup of this condition [6]. A comprehensive review of the literature from 2009 to 2019 identified only 16 patients with TC who underwent radionuclide perfusion studies, with 11 demonstrating abnormal findings and 9 showing apical perfusion deficits [5]. This paucity of cases emphasizes that nuclear imaging is infrequently performed during the acute presentation of TC and even more rarely serves as the primary diagnostic modality.

According to the International Takotsubo Diagnostic Criteria (InterTAK), the diagnosis of TC requires demonstration of transient regional wall motion abnormalities extending beyond a single epicardial coronary artery distribution, typically in a circumferential pattern, combined with the absence of culprit atherosclerotic coronary artery disease [7]. Echocardiography is critical to the diagnosis, but cannot conclusively differentiate TC from ischemic cardiomyopathy [8]. Echocardiography will demonstrate hypokinesis of the apical segments, with preservation of contractility in the basal segments. This pattern noted on echocardiography can also be seen with patients who have a high-grade stenosis involving the left anterior descending artery that reaches the cardiac apex. 

The rarity of TC diagnosis on nuclear imaging relates to several factors. First, echocardiography is typically the first-line imaging modality in the acute setting due to its availability, portability, and ability to rapidly assess wall motion abnormalities and complications [6, 9]. Second, the diagnostic criteria for TC emphasize the demonstration of characteristic wall motion patterns and exclusion of obstructive coronary disease through angiography, with nuclear imaging not included as a required component [7]. Emerging nuclear imaging techniques, including hybrid PET/SPECT and novel radiotracers, may further improve diagnostic accuracy and patient outcomes, supporting the integration of nuclear imaging into standard diagnostic protocols for suspected TC [3-5, 10]. However, given the current evidence base and established diagnostic algorithms, nuclear imaging remains an adjunctive rather than primary diagnostic tool, with its greatest value in cases where traditional imaging modalities yield equivocal results or when detailed pathophysiologic characterization is desired for research purposes.

## Conclusions

Takotsubo cardiomyopathy is a reversible syndrome that can clinically mimic ACS. Our patient was able to be diagnosed with TC with the use of non-invasive imaging, then confirmed with invasive coronary angiography. Her left ventricular ejection fraction recovered after three months, and she returned to her baseline function. This case highlights the potential role of nuclear MPI in identifying the characteristic perfusion pattern of TC, which can support its inclusion in diagnostic algorithms. Greater awareness and use of nuclear imaging in patients with acute chest symptoms can improve diagnosis and guide appropriate management, ultimately enhancing patient care.
